# *In vitro* effect of TiF_4_/NaF solution on the development of radiation-induced dentin caries

**DOI:** 10.1590/1678-7757-2024-0024

**Published:** 2024-06-10

**Authors:** Beatriz Martines de SOUZA, Eduardo Lista FRANCISCO, Aline Silva BRAGA, Paulo Sergio da Silva SANTOS, Marilia Afonso Rabelo BUZALAF, Ana Carolina MAGALHÃES

**Affiliations:** 1 Universidade de São Paulo Faculdade de Odontologia de Bauru Departamento de Ciências Biológicas Bauru São Paulo Brasil Universidade de São Paulo, Faculdade de Odontologia de Bauru, Departamento de Ciências Biológicas,Bauru, São Paulo, Brasil.; 2 Universidade de São Paulo Faculdade d e Odontologia de Bauru Departamento de Cirurgia, Estomatologia, Patologia e Radiologia Bauru São Paulo Brasil Universidade de São Paulo, Faculdade d e Odontologia de Bauru, Departamento de Cirurgia, Estomatologia, Patologia e Radiologia, ,Bauru, São Paulo, Brasil.

**Keywords:** Dental caries, Fluorides, Biofilms, Radiotherapy

## Abstract

**Objective:**

To evaluate the protective effect of an experimental solution containing TiF_4_/NaF on the development of radiation-induced dentin caries lesions.

**Methodology:**

bovine root samples were irradiated (70Gy) and distributed as following (n=12/group): Commercial Saliva (BioXtra), NaF (500 ppm F-), TiF_4_ (500 ppm F), TiF_4_/NaF (TiF_4_: 300 ppm F-, NaF: 190 ppm F-), and Phosphate buffer solution (PBS, negative control). Biofilm was produced using biofilm from irradiated patients and McBain saliva (0.2% of sucrose, at 37oC and 5% CO_2_) for five days. The treatments were applied 1x/day. Colony-forming units (CFU) were counted and demineralization was quantified by transversal microradiography. The ANOVA/Tukey test was applied for all parameters.

**Results:**

All treatments reduced CFU for total microorganisms. TiF_4_ reduced *Lactobacillus sp*. (7.04±0.26 log10 CFU/mL) and *mutans streptococci* (7.18±0.28) CFU the most, when compared to PBS (7.58±0.21 and 7.75±0.17) and followed by NaF (7.12±0.31 and 7.34±0.22) and TiF_4_/NaF (7.16±0.35 and 7.29± 0.29). TiF_4_ and Commercial saliva showed the lowest integrated mineral loss (ΔZ-vol%.mm) (1977±150 and 2062±243, respectively) when compared to PBS (4540±335), followed by NaF (2403±235) and TiF_4_/NaF (2340±200). Commercial saliva was the only to significantly reduce mineral loss (LD-µm) (111±25) compared to PBS (153±24).Mean mineral loss (R-vol%) decreased by 35.2% for TiF_4_ (18.2±3.3) when compared to PBS (28.1±2.9) Conclusion: TiF_4_/NaF has a comparable anti-cariogenic effect to TiF_4_ and Commercial saliva under the model in this study.

## Introduction

Radiation-related caries (RRC) refers to an adverse outcome of radiotherapy in individuals with head and neck cancer,^1^ showing high incidence in these patients, around 16% after one year of radiotherapy and 74% after seven years of treatment.^2,3^ Although dental caries is related cariogenic biofilm and a diet rich in sugars, inherited factors of the host can modulate the disease.^4^

RRC has a high and fast potential for tooth destruction with involvement of non-classical dental surfaces, such as cusps and incisal regions.^1,5^ A possible justification for these characteristics is that radiotherapy damages the salivary glands. This impairment reduces salivary flow and buffering capacity and alters the concentrations of antimicrobial electrolytes and proteins.^1,5,6^ Furthermore, difficulties in oral hygiene due to the complications of radiotherapy (such as mucositis) and change in diet to pastier and carbohydrate rich-foods can contribute to the development of the disease.^1,5-7^

Thus, the high incidence of RRC necessitates guidance regarding diet and good mechanical oral hygiene by patients.^5,8^ This care must be associated with the use of oral antiseptics, artificial saliva, and fluorides to improve approaches to control the disease.^9^

Artificial saliva has been indicated to reduce hyposalivation symptoms.^10^ BioXtra is one of the most applied commercial saliva by patients with head and neck cancer.^11^ Its active ingredients include proteins (such as lysozyme and lactoferrin) and fluoride (sodium monofluorophosphate, 1,500 ppm F^-^). No study has evaluated the commercial saliva BioXtra for its anti-caries or/and antimicrobial effect in dentin biofilms.

The use of fluoride products is indicated for patients to reduce the progression and aggressiveness of RRC^12^ as long as hyposalivation persists or caries activity remains high.^5^ Accordingly, previous studies have shown that titanium tetrafluoride (TiF_4_) more efficiently reduces tooth demineralization when compared to sodium fluoride (NaF) varnishes or solutions.^13,14^ In a microcosm biofilm model, TiF_4_ more effectively reduced the demineralization of irradiated dentin than NaF varnish.^13^ The daily use of fluoridated solution could benefit this specific population much more than a professional application (as varnish) due to patient compliance and frequency of application.

Vertuan, et al.^14^ (2021) have recently evaluated the effects of TiF_4_/NaF solutions (with or without chitosan association) in preventing the demineralization of healthy dentin in a cariogenic microcosm biofilm model. Both solutions containing TiF_4_/NaF (with or without associated chitosan) effectively reduced the development of dentin caries in a healthy substrate.

Considering the promising result on healthy dentin and the lack of studies about TiF_4_/NaF on irradiated dentin, this research aimed to test the protective effect of an experimental solution containing TiF_4_/NaF on irradiated root dentin in a microcosm biofilm model produced from biofilm collected from patients subjected to head and neck radiation. Its null hypothesis suggests no difference between fluoride solutions and BioXtra (commercial saliva) when compared to the negative control regarding antibiofilm and protection against demineralization.

## Methodology

### Biofilm collection

The study protocol approved by the local Ethics Committee (CAAE: 97497318.00000.5417) before participants signed informed consent forms. Dental biofilm was donated by two donors (one 57 year-old woman with 24 teeth and a 65 year-old man with 20 teeth) who received total head and neck 3D radiotherapy (final dose: 70 Gy) five months before this study according to the following inclusion criteria: low non-stimulated salivary flow (<0.3 mL/min), absence of gingivitis and/or mucositis, neither using antibiotics nor having undergone professional fluoride application in the prior three months, and having at least 20 teeth)^13^. The biofilms pool was mixed in 0.9% saline solution (proportion 2 mg: 1 ml) and stored in 1 ml aliquots at −80ºC.^13,15^

### Tooth specimen preparation and treatment groups

In total, 60 bovine dentin samples were prepared (4 mm x 4 mm)^16^ after approval of the ethics committee on animal research (CEUA, Number: 004/2018). The bovine teeth, provided by Frigol S.A., underwent a single exposure to 70 Gy.^13^ The allocation proposal included average roughness (0.36±0.03 µm), using a contact profilometer (Mahr Perthometer) and the MarSurf XCR-20 software (Mahr Perthometer). Subsequently, two-thirds of the sample surface were coated with red nail polish (Love-Risqué) to facilitate subsequent analysis of tooth demineralization by transverse microradiography (TMR). Finally, the samples were sterilized via exposure to ethylene oxide.^13^

Microcosm biofilm was produced on the irradiated dentin samples using the donated biofilm as the microorganism source and treated thus (n=12/group): Commercial Saliva - BioXtra [pH 6.2 - active components: lysozyme, lactoferrin, lactoperoxidase; colostrum extract. Other ingredients: water, propylene glycol, xylitol, sodium monofluorophosphate (MFP, 1500 ppm F^-^), poloxamer 407, hydroxyethyl cellulose, aroma, Aloe Barbadensis Leaf Juice, EDTA, lactic acid, sodium benzoate, limonene, linalool, CI42090. Lifestream Pharma, Seneffe, Belgium]; NaF (pH 6.6; 500 ppm F^-^); TiF_4_ (pH 2.4; 315 ppm Ti^4^, 500 ppm F^-^); TiF_4_/NaF (pH 4.2; TiF_4_: 190 ppm Ti^4^, 310 ppm F^-^; NaF: 190 ppm F^-^, 500 ppm F); and a phosphate buffer solution (PBS) (negative control) (pH 7.1).

### Microcosm biofilm formation

The biofilm-glycerol stock was diluted in McBain artificial saliva (pH 7.0, 2.0 g/L tryptone, 2.5 g/L mucin from porcine stomach (type II), 1.0 g/L yeast extract, 2.0 g/L bacteriological peptone, 0.2 g/L KCl, 0.35 g/L NaCl, .2 g/L CaCl_2_, 0.001 g/L hemin, 0.1 g/L cysteine hydrochloride and 0.0002 g/L vitamin K1)^17^ at a ratio of 1:50 (inoculum).^13^ The microcosm biofilm was grown in 24-well plates for five days. Dentin samples were exposed to the inoculum for eight hours. The medium was then removed and replaced by McBain Saliva with 0.2% sucrosis (1.5 mL) for further 16h. From the 2^nd^ to the 5^th^ day, the medium with sucrose was replaced once a day and the plates were then incubated at 5% CO_2_ and 37°C.^13,16^ Treatments involving artificial saliva or fluoride solutions were administered daily for one minute over four days during microcosm biofilm formation.

### Biofilm was cultivated done in biological triplicates (n=4/replicate, n final=12).

#### Colony-forming unit (CFU) counting

The bacterial suspension was prepared in the NaCl solution, diluted in each well plate, and then sonicated (Sonifier Cell Disruptor B-30, Branson) for 30 s at 20 W. Bacterial suspensions were diluted to either 10^4^ or 10^-5^ and spread onto Petri dishes at 25 μL per dish. Subsequently, the dishes were incubated at 37 °C with 5% CO_2_ for 48 hours.^14,16^The three agar culture media used were:^13^ (1) brain heart infusion agar (BHI; Difco) for total microorganisms (dilution factor 10^-5^); (2) SB-20M for *mutans streptococci* (*Strep. mutans* and *Strep. sobrinus*) (dilution factor 10^-4^); and (3) MRS (Kasvi) for *Lactobacillus sp*. (dilution factor 10^-5^).^16^After the 48-hour incubation period, CFU were numbered and utilized to calculate the total CFU for each type of microorganism per group. The data were then transformed into log_10_ CFU/mL.^13^

#### Transverse microradiography (TMR) - demineralization analysis

Dentin samples underwent cleaning, transverse sectioning, and polishing to a thickness of 100-120 µm. The X-ray exposure procedure (20kV and 20 mA, Softex, Tokyo, Japan), development of glass plates, and optical microscope analysis followed the method outlined by Santos, et al.^16^ (2019) utilizing the TMR system from Inspektor Research System. Subsequently, integrated mineral loss (ΔZ, %vol. µm), average mineral loss across lesion depth (mean mineral loss, R, %vol), and lesion depth (LD, µm) were computed as described.^13^

## Statistical analysis

The data underwent statistical analysis using the GraphPad Prism 7.04 software (p<0.05). Data were subjected to normality and homogeneity tests (Brown-Forsythe and Bartlett’s tests). ANOVA/Tukey tests were applied to compare the treatments regarding CFU counting and all TMR parameters (ΔZ, R, and LD).

## Results

### CFU counting

Total microorganisms counting decreased in all treatments in comparison to the negative control (PBS), except in commercial saliva (p=0.0003). The TiF_4_ solution reduced *Lactobacillus sp.* CFU the most when compared to PBS, followed by the NaF and TiF_4_/NaF solutions, which failed to differ from each other but did so from PBS. Commercial saliva (BioXtra) resembled the NaF and TiF_4_/NaF solutions but failed to differ from PBS (p<0.0001). *Mutans streptococci* also decreased in all treatments in comparison to PBS but TiF_4_ decreased its CFU the most, significantly differing from commercial saliva as well (p<0.0001) (Table 1).

**Table 1:** Mean ± SD of the CFU count (log10 CFU/mL) for total microorganisms, *Lactobacillus sp*. (10-5), and *mutans streptococci* (10-4) of microcosm biofilm treated with different solutions

	total microorganisms	*Lactobacillus sp*.	*mutans streptococci*
Commercial Saliva (BioXtra)	7.43 ± 0.42^AB^	7.37 ± 0.35^AB^	7.45 ± 0.17^B^
NaF solution	7.35 ± 0.35^B^	7.12 ± 0.31^BC^	7.34 ± 0.22^BC^
TiF_4_ solution	7.26 ± 0.30^B^	7.04± 0.26^C^	7.18 ± 0.28^C^
TiF_4_ /NaF solution	7.24 ± 0.35^B^	7.16 ± 0.35^BC^	7.29 ± 0.29B^C^
PBS (negative control)	7.70± 0.26^A^	7.58 ± 0.21^A^	7.75 ± 0.17^A^

Different letters show statistical difference between treatments. ANOVA/Tukey: total microorganisms (p=0.0003); *Lactobacillus* spp. (p<0.0001); *S. mutan*s/ *S. sobrinus* (p<0.0001).

### TMR analysis

**Figure 1:**
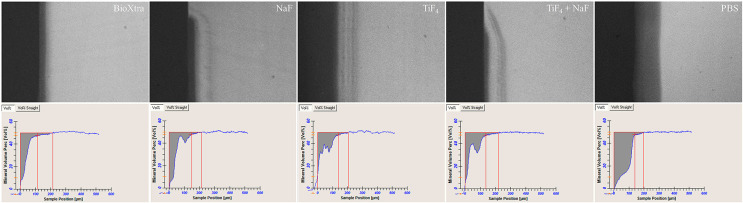
Representative TMR images and mineral profile of carious lesions under the following treatments: BioXtra; NaF solution; TiF4 solution; TiF4/NaF solution; PBS.

TiF_4_ and commercial saliva reduced integrated mineral loss (ΔZ) the most, failing to differ from each other but significantly doing so from the negative control (PBS) and NaF and TiF_4_/NaF (p<0.0001). NaF and TiF_4_/NaF solutions showed similar ΔZ values, differing from the negative control. The same results occurred for mean mineral loss (R). All treatments resembled each other as they reduced R in comparison to the negative control (PBS). Commercial saliva was the only one to significantly reduce lesion depth (LD) in comparison to the negative control (p<0.0001) (Table 2, Figure 1).

**Table 2:** Mean ± SD of the integrated mineral loss (ΔZ, vol%. μm), lesion depth (LD, μm) and mean mineral loss (R, vol%) of irradiated dentin subjected to microcosm biofilm and treated with different solutions

	DZ	LD	R
	(vol%.mm)	(mm)	(vol%)
Commercial Saliva (BioXtra)	2062 ± 243^C^	111 ± 25^B^	18.2 ± 3.3^B^
NaF solution	2403 ± 235^B^	150 ± 20^A^	15.9 ± 2.0^BC^
TiF_4_ solution	1977 ± 150^C^	143 ± 21^A^	14.3 ± 2.7^C^
TiF_4_ /NaF solution	2340 ± 200^B^	141± 13^A^	16.4 ± 1.5^BC^
PBS (negative control)	4540 ± 335^A^	153 ± 24^A^	28.1 ± 2.9^A^

Different letters show statistical difference between treatments. ANOVA/Tukey: ΔZ (p<0.0001); LD (p<0.0001); R (p<0.0001).

## Discussion

TiF_4_ and commercial saliva (BioXtra) had the best anti-cariogenic effect, followed by TiF_4_/NaF and NaF solutions. All treatments differed from the negative control in this model (Figure 1). Therefore, this research rejected its null hypothesis.

Considering the high and significant impact of RCC, patients undergoing radiotherapy in the head and neck region could benefit from the use of homecare fluoride products as fluoride increases remineralization and decreases demineralization.^12^ Patients undergoing radiotherapy should rinse with a fluoride solution before radiotherapy and for as long as symptoms of hyposalivation last as a way of controlling the emergence and progression of carious lesions.^5^

Few studies have evaluated the effect of fluoride solutions in patients undergoing radiotherapy in the head and neck region.^18,19^ A previous clinical study observed that patients who failed to adhere to any fluoride therapy after radiotherapy had a significantly greater increase in caries incidence when compared to the groups that received treatment with 1% NaF solution or SnF_2_ gel (0.4% Sn^2^).^18^ Among the different types of fluorides, SnF_2_ reduced the incidence of decayed root surfaces the most after three months of use.^18^ Another clinical study evaluated the use of 0.05% NaF fluoride solution (alone or with 0.1% chlorhexidine or ZnCl_2_) in reducing bacteria in biofilm in patients undergoing head and neck radiotherapy. The authors concluded that only by associating NaF to 0.1% chlorhexidine decreased *S. mutans* CFU, when compared to 0.05% NaF only, showing the limited effect of this salt.^19^

Fluoride varnishes have been recently tested by a microcosm biofilm model to simulate RRC.^13^ TiF_4_ varnish reduced integrated mineral loss (ΔZ) by 42% when compared to the negative control and NaF varnish. Despite its successful results, TiF_4_ varnish solutions must be applied by professionals, demand frequent visits, and incur in high costs. Thus, the use of homecare fluoride solutions containing TiF_4_ can offer an alternative and provide greater patient compliance as this study found that it reduced 48% of dentine caries.

Moreover, a microcosm biofilm model have tested TiF_4_/NaF solutions (alone or associated with chitosan) on healthy root dentin.^14^ Both solutions containing TiF_4_/NaF (alone or with chitosan) effectively reduced the development of dentin caries in a healthy substrate, decreasing ΔZ by 27% in relation to the negative control (PBS), but without any antibacterial action.^14^

All fluoride solutions in this study reduced the development of caries in irradiated dentin. While TiF_4_ reduced ΔZ by 56%, TiF_4_/NaF reached 48% in relation to the negative control (Table 2). Therefore, associating fluorides show decreased effectiveness when compared to TiF_4_ alone since these mixtures reduced the level of Ti^4^ responsible for the protective effect. Furthermore, unlike Vertuan, et al.,^13^ (2021) the tested solutions showed antimicrobial effect, reducing CFU counts by a 6% average in relation to PBS, which, in turn, may be clinically irrelevant. No previous study has explained the mechanism behind any antimicrobial effect of TiF_4_.

Despite the superior effect of the TiF_4_ solution, its low pH (2.4) makes its homecare use unfeasible. Therefore, despite a reduction in effectiveness, TiF_4_ combined with NaF in solution may be offer alternative with a favorable pH for use (4.2) and good patient acceptance.^20^

The commercial saliva BioXtra showed similar results to TiF_4_ in reducing integrated mineral loss (54%) and CFU counting for cariogenic microorganisms. Similar results occurred in irradiated enamel treated with commercial saliva BioXtra, except the lack of effect on CFU counting.^21^ Commercial saliva BioXtra is clinically applied as artificial saliva to relieve symptoms of dry mouth caused by radiotherapy.^11^It has lysozyme, lactoferrin, and lactoperoxidase, which are antimicrobial proteins.^22^ It contains MFP (1500 ppm F-); making artificial saliva able to reduce demineralization and increase remineralization. Considering the results in this study and of a previous study on enamel,^21^ the protective effect of commercial saliva BioXtra on tooth structure seems to be more relevant than antimicrobial effect on dental biofilm, which requires better explanations in the future.

The tested fluoridated solutions have 500 ppm of Fluoride, compatible with the content of commercial mouth rinses, whereas the commercial saliva BioXtra has 1500 ppm F^-^, compatible with the content of fluoridated dentifrices. Despite the one-third of F in the TiF_4_ solution, the presence of titanium induces the deposition of a glaze-like, acid resistant layer that is rich in titanium dioxide and titanium phosphate di-hydrate, providing a greater mechanical barrier than NaF.^23^ Moreover, the low pH of TiF_4_ can induce a greater deposition of fluoride on tooth structures than conventional fluorides such as NaF and MFP.^23,24^ The mechanism of action of conventional fluorides acts by forming a CaF_2_-like layer that acts as a mechanical barrier and as a source of fluoride during acid challenges, offering less acid resistance than the glaze-like layer formed from TiF_4_.^23,25^ The combination of TiF_4_ with NaF may enable both mechanisms of action against tooth demineralization.

This study, despite trying to simulate the oral environment, is incomparable to *in vivo* models in terms of evidence since it found no variation in patients undergoing clinical trials. Still, the effect of TiF_4_ associated or not to NaF as a mouth rinse showed some benefits in reducing dentin demineralization in a model simulating radiation-induced caries lesion. If clinically confirmed, this result will greatly aid patients with oral cancer undergoing radiotherapy.

## Conclusion

Although the protective effect of the TiF_4_/NaF was inferior to that of the pure TiF_4_ solution in this experimental model, future clinical trials could consider this new experimental solution as a substitute since it can be commercialized due to its pH. It would be interesting to carry out clinical trials comparing TiF_4_/NaF solutions and the commercial saliva BioXtra (associated or not) for their anti-caries efficacy, cost, and patient acceptability since, in principle, they have different proposals for clinical use and could even be applied in combination.
